# Assessing the Quality of Solvent-Assisted Lipid Bilayers Formed at Different Phases and Aqueous Buffer Media: A QCM-D Study

**DOI:** 10.3390/s24186093

**Published:** 2024-09-20

**Authors:** Marta Lavrič, Laure Bar, Martin E. Villanueva, Patricia Losada-Pérez, Aleš Iglič, Nikola Novak, George Cordoyiannis

**Affiliations:** 1 Condensed Matter Physics Department, Jožef Stefan Institute, 1000 Ljubljana, Slovenia; marta.lavric@ijs.si (M.L.); nikola.novak@ijs.si (N.N.); 2 Laboratory of Physics, Faculty of Electrical Engineering, University of Ljubljana, 1000 Ljubljana, Slovenia; ales.iglic@fe.uni-lj.si; 3 Experimental Soft Matter and Thermal Physics Group, Department of Physics, Université Libre de Bruxelles, 1050 Brussels, Belgium; martin.villanueva@ulb.be (M.E.V.); patricia.maria.losada.perez@ulb.be (P.L.-P.)

**Keywords:** supported lipid bilayers, solvent-assisted lipid bilayers, vesicle adsorption fusion and rupture, quartz crystal microbalance with dissipation monitoring

## Abstract

Supported lipid bilayers (SLBs) are low-complexity biomimetic membranes, serving as popular experimental platforms to study membrane organization and lipid transfer, membrane uptake of nanoparticles and biomolecules, and many other processes. Quartz crystal microbalance with dissipation monitoring has been utilized to probe the influence of several parameters on the quality of SLBs formed on Au- and SiO_2_-coated sensors. The influence of the aqueous medium (i.e., buffer type) and the adsorption temperature, above and below the lipid melting point, is neatly explored for SLBs of 1,2-dimyristoyl-sn-glycero-3-phosphocholine and 1,2-dipalmitoyl-sn-glycero-3-phosphocholine formed by a solvent exchange. Below the lipid melting temperature, quality variations are observed upon the formation on Au and SiO_2_ surfaces, with the SLBs being more homogeneous for the latter. We further investigate how the buffer affects the detection of lipid melting in SLBs, a transition that necessitates high-sensitivity and time-consuming surface-sensitive techniques to be detected.

## 1. Introduction

Biological membranes constituting the cell and organelle boundaries perform multiple functions, allowing ions and nutrients to enter and, at the same time, acting as barriers against toxic substances. They are composed of a lipid bilayer bearing a large number of phospholipids and glycolipids, as well as embedded and peripheral proteins. Cholesterol, interspersed within the lipid bilayer, modifies locally the membrane fluidity by forming so-called rafts that are important for cell signaling and trafficking [1,2,3,4,5]. Overall, the biological membrane is highly complex [6,7,8,9,10,11] and thus model membranes, so-called biomimetic membranes, are frequently chosen as experimental testbeds. The latter have fewer types of constituent units than their biological counterparts, and their composition and geometry can be precisely controlled. Supported lipid vesicles (SLVs) and supported lipid bilayers (SLBs) represent typical examples of biomimetic membranes [12,13]. Although the solid substrate affects, to a certain degree, the properties of the supported membranes, it has the major advantage of enabling their study by means of surface-sensitive techniques, such as atomic force microscopy (AFM) [14] and quartz crystal microbalance with dissipation monitoring (QCM-D) [15,16,17].

QCM-D is a highly sensitive and versatile experimental technique, using the acoustic waves generated by the oscillation of a piezoelectric crystal quartz sensor to monitor mass variations, down to only a few ng/cm^2^, at the sensor-sample interface [15,18]. It possesses certain advantages, such as measuring molecular events in real-time as well as being a label-free (no need to probe molecules or fluorescent agents that could influence the sample’s inherent properties) and non-destructive method. The variations of two quantities are recorded in real-time: (i) the sensor’s oscillation frequency, related to the uptake or loss of mass, and (ii) the dissipation factor, related to the viscoelastic properties of the adsorbed mass. Various coatings can be applied on the quartz sensor’s surface and serve as substrates for SLBs and SLVs, tailored to the specific needs of each experiment. QCM-D is extensively used in chemical- and bio-sensing [19,20,21] and in studying lipid phase transitions [22,23]. It can additionally probe the interaction with or the inclusion of nanoscale entities, such as vesicles, nanoparticles, and quantum dots, within the lipid bilayer [24,25,26,27]. QCM-D data are analyzed with different models to provide the thickness and the viscoelastic properties of adsorbed films [15,28,29]. They are very often combined with AFM and calorimetry results to provide a multiple-scale characterization of lipid membranes [30,31,32].

The most common and well-established method to create SLBs is vesicle adsorption, fusion, and rupture (VAFR) [33]. In this approach, lipid vesicles are deposited on a very hydrophilic substrate, and due to their strong adhesion on the surface, they deform, fuse, and finally rupture. The so-produced SLBs are of good quality, spreading and covering the surface homogeneously. The main drawback of VAFR is that it is limited to very hydrophilic surfaces, which leads to the rupture of vesicles with low bending modulus. In a few past studies, researchers exploited the idea of gradually exchanging an organic solution of lipid monomers with a buffer to form SLBs [34,35]. This was more systematically investigated and optimized by Tabaei et al. [36,37], who introduced the term solvent-assisted lipid bilayers (SALBs). This method consists of three well-defined steps: (i) dissolution of the lipid in an organic solvent (e.g., methanol, ethanol, or isopropanol), which is water-miscible, (ii) gradual exchange of the organic solvent with a buffer, and (iii) formation of SLBs in the final equilibrated state. Ferhan et al. [38] have provided a thorough description of all SALB steps and many useful tips, including the optimal lipid concentration and flow rates and the choice of organic solvent depending on the type of lipid. The advantages of this method are the short-time scale required for sample preparation (faster than VAFR, which requires the extrusion of vesicles), the independence of the substrate’s surface energy, and the good quality of the so-produced SLBs. SALBs have been chosen in many studies performed on flat and topographically complex surfaces where VAFR fails to form SLBs [39,40,41,42,43] and is extended to create hybrid lipid-polymer [44] and polymer bio-interfaces [45]. A similar yet alternative approach, referred to as a chaotropic agent-assisted supported lipid bilayer, has also been recently proposed, bypassing the use of organic solvents during SLB formation [46]. Several studies have reported on the quality of the SALBs by varying the different parameters. Tabaei et al. [36] have compared SALBs in the liquid phase of the zwitterionic lipid 1,2-dioleoyl-sn-glycero-3-phosphocholine on SiO_2_, Au and alkanethiol-treated Au surfaces. Tabaei et al. [37] investigated the SALBs of charged lipids on SiO_2_ and Al_2_O_3_. The same authors also studied the influence of the (zwitterionic) lipid membrane state on SALBs formed on SiO_2_ surfaces and evidenced negligible posterior adsorption of bovine serum albumin protein in so-produced bilayers irrespective of the state [39]. In a spectroscopic study, Hohner et al. [35] presented phase diagrams of ternary systems, composed of lipid, organic solvent, and water, as a function of temperature and water content. Moreover, Betlem et al. [42] explored the influence of the initial buffer concentration (i.e., starting from the dissolution of the lipid in a mixture of buffer and organic solvent) on the SALB of 1,2-dimyristoyl-sn-glycero-3-phosphocholine (DMPC).

Apart from being much less sensitive to the surface energy of solid surfaces, SALBs are apparently less dependent on the phase at which the lipid is dissolved [39]. This is an additional advantage as compared to VAFR, which relies on vesicle rupture and only works for lipids in its liquid-disordered phase, a phase with a bending modulus an order of magnitude smaller than the gel phase [47]. This advantage has, however, been much less explored than the effect of surface energy. As a matter of fact, a systematic comparison of SALBs performed at temperatures corresponding to different lipid phases (e.g., liquid-disordered versus gel-ordered), on Au and SiO_2_ simultaneously, and using three different buffers has not yet been carried out to the best of the authors’ knowledge.

In this work, we report on the influence that several parameters exert on the quality of SLBs, using two common zwitterionic lipids: DMPC and 1,2-dipalmitoyl-sn-glycero-3-phosphocholine (DPPC). Through QCM-D frequency and dissipation shifts, we assess the influence of the adsorption temperature (above or below the lipid melting temperature *T*_m_) and the type of aqueous buffer medium on the quality of SALBs on SiO_2_. In one case, we incorporate into the aqueous medium trisethylenediaminetetra-acetic acid (EDTA), a frequently used additive in solutions for biomembrane studies. EDTA has been reported to impact the organization of charged lipid- [48] and zwitterionic lipid layers [49], and its effect on SALBs has not yet been explored. We further assess how the choice of buffer influences the detection of the lipid melting transition. Finally, we perform a brief comparison of SLBs produced by VAFR and SALBs [38,50].

## 2. Materials and Methods

### 2.1. Materials

The lipid compounds DMPC and DPPC have been purchased from Avanti Polar Lipids in powder form. They have been constantly stored at −20 °C and kept only for short time periods at room temperature during the weighing process for sample preparation. Three buffers, namely, 2-[4-(2-hydroxyethyl)piperazin-1-yl]ethanesulfonic acid (HEPES), tris(hydroxymethyl)aminomethane (Tris), and Tris-EDTA were prepared using high-purity HEPES powder ≥ 99.8%, Trizma base powder ≥ 99.9%, NaCl ≥ 99%, all purchased from Sigma Aldrich (Steinheim, Germany), and EDTA powder from VWR Chemicals (Leuven, Belgium).

The HEPES buffer solution (pH 7.4) was prepared by dissolving 10 mM HEPES and 150 mM NaCl in water. The Tris buffer solution (pH 7.4) was prepared by dissolving 10 mM Trizma base and 150 mM NaCl in water. The Tris-EDTA buffer solution (pH 7.4) was prepared by dissolving 10 mM Trizma base, 150 mM NaCl, and 5 mM EDTA in water. Freshly purified MilliQ water with a resistivity of 18.2 MΩ·cm at 25 °C (Simplicity^®^ purification system, Merck Millipore, Overijse, Belgium) was used in all cases. For every buffer, the pH was adjusted to 7.4 by adding a tiny volume of either HCl 37% solution (Avantor Performance Materials, Gliwice, Poland) or 1 M NaOH solution (prepared with NaOH pellets from VWR Chemicals, Leuven, Belgium) and tracked with a Metrohm 827 lab pH-meter (Herisau, Switzerland). The buffer solutions were filtered through 0.2 µm-pore size PES membranes (Corning GmbH—Wiesbaden, Germany) and stored at 4 °C until being used. The extrusion of lipid suspensions was performed with a mini-extruder kit from Avanti Polar Lipids (Alabaster, AL, USA), using polycarbonate membranes and filter supports from Cytiva Whatman^TM^ (Maidstone, UK).

### 2.2. Lipid Sample Preparation

For the VAFR process, dispersions of unilamellar lipid vesicles were produced as follows: DPPC and DMPC powders were first dissolved in high-purity (>99.9%) chloroform. The mixture was slowly dried under a continuous mild flow of Ar to form lipid films, which were vented overnight to eliminate any chloroform residues. The dried films were then hydrated with fresh buffer to a concentration of 2 mg/mL under magnetic stirring for 45 min. The hydration was performed by immersing the sample vials in a temperature-controlled water bath, at *T* = 60 °C for DPPC and *T* = 40 °C for DMPC samples. In each case, the hydration was carried out well above the melting temperatures of DPPC (*T*_m_ ~ 42 °C [51]) and DMPC (*T*_m_ ~ 24 °C [52]) to obtain large multilamellar vesicles of different sizes. These vesicles were subsequently extruded through membranes with a pore size of 50 nm, using a series for 25 passes, to become unilamellar. The extruded solutions were finally diluted in the buffer to obtain a working concentration of 0.5 mg/mL.

For the SALB process, lipids were injected in monomer form since no vesicle formation is necessary in this case. Prior to injection, the lipid powder was dissolved at a concentration of 0.5 mg/mL in high-purity (>99.9%) isopropanol (IPA), which among other organic solvents (ethanol, methanol, etc.) yields the best-reported results [38]. The solution (lipid in IPA) was sonicated for 5 min just before injection into the QCM-D modules.

### 2.3. Experimental Setup

A QSense Analyzer (Biolin Scientific, Gothenburg, Sweden) QCM-D apparatus has been employed for all measurements. It is characterized by a high sensitivity, down to a few ng per cm^2^. It enables simultaneous monitoring of frequency and dissipation changes, Δ*f*_n_/n and Δ*D*, where *n* is the overtone number; the fundamental frequency is 5 MHz. A QSense Analyzer allows the simultaneous execution of four experiments. The sample temperature can be changed in the range from 15 °C to 60 °C and with a precision of ±0.01 °C, allowing heating and cooling ramping with variable scanning rates. Two types of sensors have been used for the present measurements: Au-coated (QSX301) and SiO_2_-coated (QSX303), purchased from Biolin Scientific, and cleaned following standard protocols. Before each experiment, the sensors were exposed to UV light using a UV-ozone cleaner for 15 min. The changes in Δ*f*_n_/n and Δ*D* were monitored for four different overtones (from 3rd to 9th). The bilayers were formed at either 15 °C or 52 °C., i.e., being simultaneously either above or below the melting temperatures of both lipids [51,52]. The layer formation processes were followed by heating and cooling cycles between 15 °C to 52 °C to detect lipid phase transitions.

**Figure 1:**
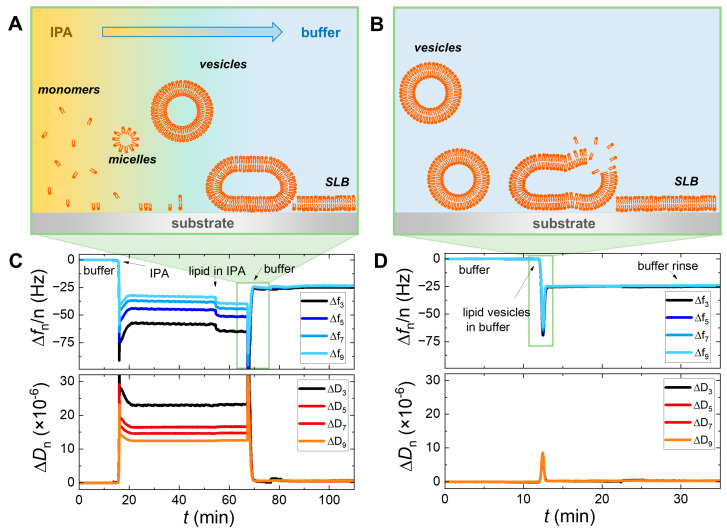
The QCM-D sensing and schematic representations of the lipid bilayer formation for the SALB (**A**,**C**) and VAFR (**B**,**D**) methods. The lower panels (**C**,**D**) display typical QCM-D signal responses for a DPPC bilayer formation performed on SiO_2_ substrate at 52 °C, using HEPES as a buffer, via the SALB and the VAFR methods, respectively. The frequency (Δ*f*) and dissipation (Δ*D*) shift from the 3rd to the 9th overtones are presented. Panels (**A**,**B**) are explanatory schemes of the events occurring during the corresponding steps highlighted in the green squares.

For the SALB formation, experiments were carried out with DMPC and DPPC on SiO_2_ or Au, using the three different types of buffers. The process can be followed in Figure 1A,C, which show a schematic representation and the QCM-D frequency and dissipation variations along each step of SALB formation at 52 °C on SiO_2_. First, a baseline with pure buffer was established for approximately 10 min. Second, the buffer was gradually exchanged with pure IPA until a complete solvent exchange took place and a new stable baseline was achieved. As can be seen in Figure 1C, the change of the aqueous medium causes a frequency decrease and an overtone spreading, which do not affect the final SLB. Afterward, the lipid dissolved in IPA at 0.5 mg/mL concentration was injected for 15 min, before a new buffer replacement. The final plateau values of frequency Δ*f* and dissipation Δ*D* shifts are related solely to the mass of the formed SLB. All the injections were performed with a flow rate of 100 μL/min. The values reported in the manuscript are averages and standard deviations of different experiments of the same system.

Figure 1B,D display the process of VAFR for the formation of a DPPC bilayer performed on SiO_2_, at 52 °C. In this case, a baseline with pure buffer (HEPES) was first established. The suspension of (extruded) 50 nm-lipid vesicles (0.5 mg/mL in buffer) was then injected into the QCM-D chamber. The vesicle adsorption leads to a frequency decrease (Δ*f* < 0) and a dissipation increase (Δ*D* < 0), reflecting the accumulation of vesicles onto the surface and the formation of a viscous SLV. At a certain coverage density on the sensor surface, the vesicles fuse and finally rupture to form a bilayer. The buffer and lipid release occurring during this step is accompanied by an increase in the frequency and a decrease in the dissipation, marking the formation of an organized lipid layer. After reaching a stable signal plateau, a rinsing step with buffer was performed to remove any remaining vesicles from the chamber. All injections were carried out at a flow rate of 50 μL/min. The calculated hydrated masses per unit area were obtained using two models provided by QSense DFind 1.3.0 software. The composite Sauerbrey model was chosen for rigid organized lipid bilayers, using a weighted average of all harmonics for the fit. The Dfind broadfit model was chosen for soft, non-homogeneous lipid layers, fitting all points independently to obtain the best match. For configuring modeling at 15 °C, the Dfind “fat” (density of 910 g/L) was set as the default layer material, and the “buffer-15 °C” (density of 1007 g/L) was set as the baseline default bulk liquid.

## 3. Results

In this section, the experimental results are presented, focusing on SALB formation on different surfaces (SiO_2_ and Au), adsorption temperatures (in the liquid-disordered and in the gel-ordered phases), and buffers (HEPES, Tris, Tris-EDTA). DPPC and DMPC lipids have been used to probe the differences in SALB quality. We mostly present DPPC results accompanied by DMPC ones in some cases; detailed DMPC figures are shown in Figures S1 and S2 of the Supplementary Material.

**Figure 2:**
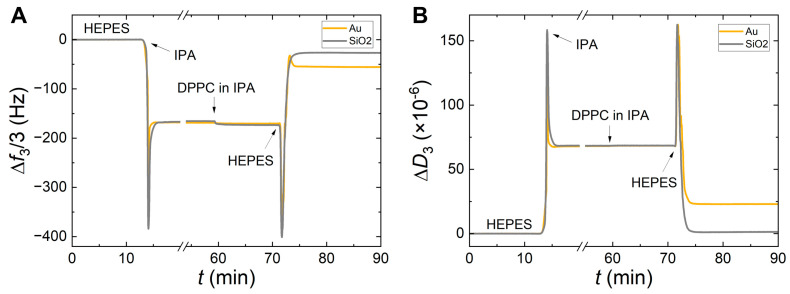
The influence of the solid surface on a DPPC SALB, performed at 15 °C is shown here for Au (yellow curves) and SiO_2_ (grey curves). The frequency and the dissipation changes of the 3rd overtone are shown for both cases on the left panel (**A**) and the right panel (**B**), respectively.

**Figure 3:**
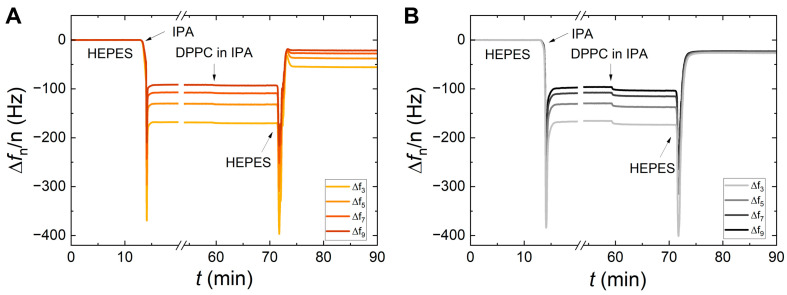
The frequency shifts for the overtones *n* = 3, 5, 7, and 9 are shown for DPPC (in HEPES) SALBs, performed at 15 °C, on Au (left panel (**A**)) and SiO_2_ (right panel (**B**)), respectively. In the case of Au, a split of the overtones is observed at the final plateau values, whereas in the case of SiO_2,_ the values are almost identical.

Figure 2 shows the DPPC SALB process on SiO_2_ and Au substrates, performed at 15 °C, well below the main transition of DPPC. The frequency (Figure 2A) and dissipation (Figure 2B) changes are very similar in both cases for the initial steps (injection of pure IPA, subsequent injection of DPPC in IPA, and IPA exchange with HEPES) and can be explained by the change of the physicochemical properties (medium density and viscosity) of the chamber environment when exchanging aqueous and non-aqueous media. However, the final plateau values are quite different for both frequency and dissipation. In the case of SiO_2_, one obtains a frequency shift of around −25 Hz and dissipation change below 10^−6^ characterizing a good-quality, homogeneous bilayer [37,53,54,55]. In the case of Au, the final frequency shift (for the 3rd overtone) of −55 Hz and at least an order of magnitude higher dissipation change of ~2 × 10^−5^ (compared to SiO_2_), mark an inhomogeneous DPPC-supported bilayer that very likely contains several patches and a few non-ruptured vesicles. Similar differences have been obtained for DMPC, depicted in Figure S1 of the Supplementary Material. The way overtones spread can be used to evaluate the homogeneity of a SLB. Figure 3 presents the frequency shifts of four different overtones (*n* = 3, 5, 7, and 9) for the DPPC SALB commonly shown in Figure 2A. In Figure 3A, assigned to the Au-coated quartz QCM-D sensor, the large split between different overtones demonstrates the deviation from a homogeneous SLB. The lower surface energy of Au (its polar component being half that of SiO_2_ [56]) allows some of the structures (e.g., vesicles) formed in the last step of the process to adsorb and stay intact. This, in turn, leads to a viscoelastic film and the response of the system is overtone-dependent. On the contrary, in Figure 3B, assigned to the SiO_2_-coated sensor, there is essentially no overtone dispersion indicating the presence of a single homogeneous bilayer on the surface.

It is worth noting a tiny yet interesting feature observed in Figure 2 and Figure 3: when DPPC in IPA is injected, a very small frequency shift is observed. This shift is more pronounced in the case of SiO_2_ (ranging from 6 to 8 Hz) compared to Au (smaller than 2 Hz), in full agreement with a previous QCM-D study [36]. This difference between SiO_2_ and Au is repeatedly observed for different lipids (DPPC, DMPC, and DOPC), aqueous buffers (HEPES, Tris), and adsorption temperatures (corresponding to liquid and gel phases), in the present study, as well as by Tabaei et al. [36]. Therefore, it is reasonable to assume that the initial lipid adsorption when dissolved in IPA depends on the surface energy of the substrate. The higher surface energy of SiO_2_ apparently enables a small (yet larger than in the case of Au) initial lipid adsorption when the lipids are in IPA, i.e., prior to the final step of exchanging IPA with a buffer where the major part of adsorption occurs. This, in turn, likely favors a more homogeneous surface coverage during the solvent exchange.

Specific models using the frequency and dissipation shifts can be applied to obtain additional information, such as hydrated masses per unit area. The Sauerbrey model is typically used for homogeneous, very thin, rigid layers, displaying overlapping frequency overtones and very small dissipation shifts (ΔD ~ 0). In our case, this model is applicable for DPPC and DMPC bilayers formed on SiO_2_ substrates. On the contrary, for SLBs formed on Au surfaces, a viscoelastic model is used since the films are softer and the overtones are spread. The calculated hydrated masses per unit area using the aforementioned models are presented in Table S1 of the Supplementary Material.

**Figure 4:**
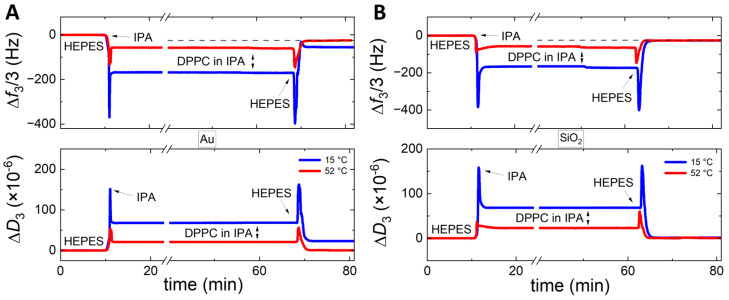
The influence of adsorption temperature on a DPPC SALB for Au (left panels (**A**)) and SiO_2_ (right panels (**B**)) substrates; the frequency and dissipation changes are shown for the 3rd overtone at the top and bottom layers of each panel, respectively. Data obtained at 15 °C are represented in blue, whereas the ones at 52 °C are in red. The dashed lines serve as guides to the eye, denoting the QCM-D frequency shifts corresponding to a complete and homogeneous SLB surface coverage [36,54].

To assess the influence of the adsorption temperature, the DPPC SALB process has been additionally performed at 52 °C. The results of the SALB performed at both phases (gel-ordered phase at 15 °C and liquid-disordered phase at 52 °C) are presented in Figure 4 for both types of substrates. As can be observed, when lipids are dissolved in their liquid-disordered phase, the final Δ*f/n* and Δ*D* plateaus approach more the established values [53,54] for complete and homogeneous SLBs. Figure 4A shows that the adsorption at 52 °C, i.e., well inside the liquid-disordered phase, improves remarkably the quality of the SALB in the case of the Au surface. On average, the shifts obtained on Au at 52 °C, Δ*f* = −26.1 ± 0.2 Hz, and Δ*D* = (0.5 ± 0.1) × 10^−6^, resemble the ones obtained on SiO_2_ with a higher surface energy (Δ*f* = −22.2 ± 0.7 Hz and Δ*D* = (0.8 ± 0.1) × 10^−6^ at 15 °C; Δ*f* = −24.9 ± 0.5 Hz and Δ*D* = (0.6 ± 0.1) × 10^−6^ at 52 °C). Analogous conclusions are drawn in the case of DMPC, the results of which are reported in Figure S2 of the Supplementary Material.

Tabaei et al. [39], in their study of DPPC and DMPC SALBs on SiO_2_, have associated the differences in the adsorbed mass with differences in packing area per molecule in the liquid and gel phases, reporting a reasonable agreement. In the present work, we systematically observe slightly larger (yet always in the range of 10^−6^) dissipation shifts when the adsorption takes place at 15 °C, indicating a somewhat lower homogeneity of the SLBs formed at the gel phase. Therefore, such a quantitative comparison as in Ref. [39] cannot be performed since it would make sense only between SLBs displaying similar surface coverage and homogeneity. Note that, as pointed out by Keller and Kasemo [54], these calculations have to be taken with caution since they neglect the hydration of lipid heads as well as the mass of the thin aqueous layer between the SLB and the substrate.

**Figure 5:**
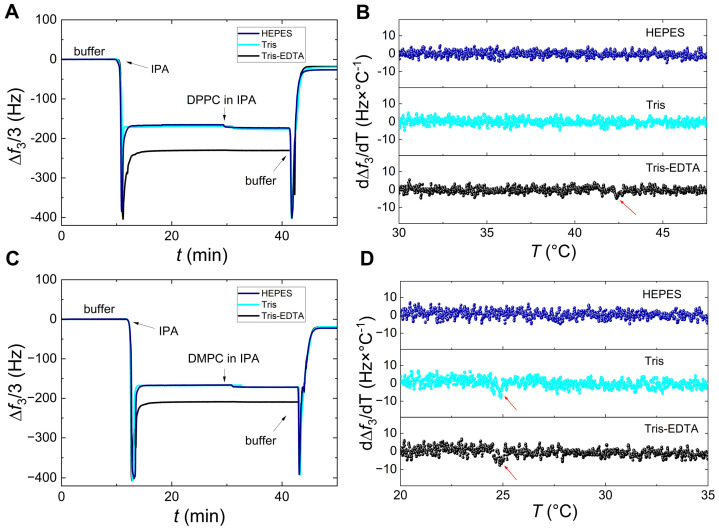
The influence of three different buffers (HEPES, TRIS, TRIS/EDTA) on the SALB process and the melting phase transition of DPPC and DMPC. The left panels show the QCM-D frequency response (3rd overtone) along the SALB formation of DPPC (panel (**A**)) and DMPC (panel (**C**)) on SiO_2_. The right panels show the temperature derivatives of frequency changes (3rd overtone) upon heating the DPPC (panel (**B**)) and DMPC (panel (**D**)) bilayers. Heating runs were performed with a scanning rate of 0.4 °C/min. The data obtained using HEPES, Tris, and Tris-EDTA buffers are represented in blue, cyan, and black, respectively. The red arrows mark the main transition peak of the lipid melting when visible (~42.5 °C for DPPC and ~24.5 °C for DMPC).

Figure 5 shows the SALB formation of DPPC (Figure 5A) and DMPC (Figure 5C) at 15 °C. These SALBs are associated with their temperature derivatives of frequency changes (3rd overtone) occurring upon subsequent heating runs for DPPC (Figure 5B) and DMPC (Figure 5D). As can be seen in Figure 5A,C, the final frequency shifts at the plateau (shown here for the 3rd overtone) are similar and, thus, almost independent of the buffer type. In particular, the frequency shifts are around −20 Hz (±2 Hz), whereas the dissipation is in all cases below 10^−6^. These values are in agreement with the formation of vesicle-free, good-quality bilayers. The same experiments performed on SiO_2_, but at 52 °C, yielded frequency shifts of −23 Hz (±2 Hz), very close to the reported values for SLBs in the literature [54,55].

After the SALB formation at 15 °C, the chambers containing the sensors and samples were heated, at a rate of 0.4 °C/min, to determine the lipid melting phase transition temperatures. The resonance frequency and dissipation of the QCM-D sensors change gradually and continuously with temperature. However, when a phase transition occurs at the sensor-sample interface, it induces an abrupt change, namely, a discontinuous jump in the resonance frequency, the temperature derivatives of which allow us to determine the phase transition peaks. For SLVs and very dissipative layers, the peak intensity can be very high. For thin and less dissipative layers, such as SLBs, the detection of phase transition is more difficult. Although no significant differences are observed in the frequency and dissipation shifts during SALB formation, the detection of the main transition of lipid melting strongly depends on the type of buffer. Figure 5B (DPPC) and Figure 5D (DMPC) show that the melting could not be detected in the case of HEPES within our QCM-D resolution. However, it is visible in the case of Tris for DMPC and in the case of Tris-EDTA for both lipids. The detection is slightly pronounced with Tris-EDTA and notably reproducible during subsequent cooling of the SALB formed at 15 °C as well as during heating of the SALB formed at 52 °C (with the same rate of 0.4 °C/min).

## 4. Discussion

**Figure 6:**
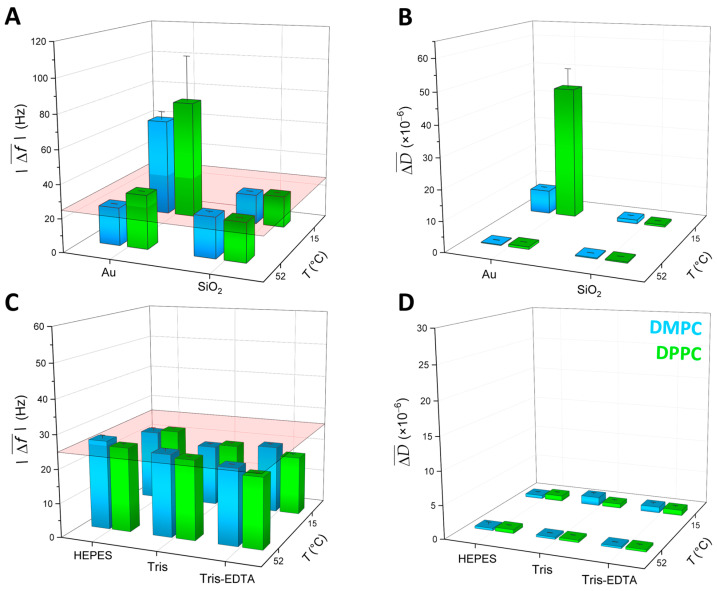
Comparative bar charts of the QCM-D average (overtones *n* = 3, 5, 7, and 9) values of the frequency and dissipation shifts occurring during the DPPC and DMPC SALB formation. The values are shown as a function of the adsorption temperature and the substrate (top panels (**A**,**B**)) for the same buffer (Tris) and as a function of the adsorption temperature and the type of buffer (bottom panels (**C**,**D**)) for the same surface (SiO_2_). In all panels, DPPC values are represented in green and DMPC ones in blue. The colored planes in panels (**A**,**C**) serve as guides to the eye, denoting the average values, as widely accepted in the literature [54,55], for a complete and homogeneous SLB.

The results presented in the previous section, as well as in the Supplementary Material, show clearly that the quality of DPPC and DMPC SALB formation is affected by several experimental parameters, such as the substrate surface, the adsorption temperature, and the buffer type. In the following, we attempt an overall evaluation and correlation among these parameters. The trends in the QCM-D average frequency and dissipation shifts obtained for DPPC and DMPC SALBs are depicted in the three-dimensional bar charts of Figure 6. One can visualize the results obtained for both lipids: (i) as a function of the substrate surface and the adsorption temperature (Figure 6A,B) when using the same buffer (Tris), and (ii) as a function of the aqueous buffer medium and the adsorption temperature (Figure 6C,D) when using the same substrate (SiO_2_). For each experiment, the mean value of four overtones (*n* = 3, 5, 7, and 9) was calculated, whereas the plotted values represent an average of the mean values of two (independent) experiments. Using this approach, the displayed error bars also illustrate the overtones spreading, allowing one to visualize the surface inhomogeneity. For display purposes, to avoid inverted bar charts, the absolute values of the frequency shifts are plotted.

**Adsorption temperature and surface impact on SALBs**. When performing the SALB process on Au-coated sensors and using Tris buffer, both frequency and dissipation shifts are much larger (as seen in Figure 6A,B) for a SALB at 15 °C compared to 52 °C. Such frequency and dissipation values mark the presence of inhomogeneity, patches, and few remaining (i.e., non-ruptured) vesicles, and the effect is more pronounced in the case of DPPC (a very large dissipation is seen in Figure 6B). On the contrary, for SiO_2_, there is a much weaker dependence on the adsorption temperature, with the frequency and dissipation values being close to each other. The best results are obtained for the adsorption on SiO_2_ at 52 °C, where average frequency and dissipation shifts of −23.3 ± 0.3 Hz and (0.4 ± 0.1) × 10^−6^ are obtained for DPPC and −24.3 ± 0.8 Hz and (0.3 ± 0.1) × 10^−6^ for DMPC, corresponding to the formation of a good-quality SLB [36,54]. The results obtained in Tris, are similar to the ones in the case of HEPES (presented in Figure 2, Figure 3 and Figure 4). At low temperatures corresponding to the gel phase and on Au substrates, the establishment of homogeneous bilayers is harder to achieve since any structures (e.g., vesicles) formed during the solvent exchange are too stiff to break and reorganize on the surface. A larger dissipation is also observed for DPPC compared to DMPC (Figure 4B and Figure S2B). At 15 °C, DMPC is closer to its melting point compared to DPPC, and some defects, in particular, non-ruptured DMPC vesicles deform more than DPPC ones, yielding smaller dissipation values.

The frequency shifts and dissipation values of our study for DPPC and DMPC adsorbed on SiO_2_ at 52 °C are in good agreement with the ones of Tabaei et al. [39], obtained in Tris at slightly different temperatures, albeit in the same (liquid-disordered) phase. A few differences, observed at lower temperatures between the values reported in the two studies, could be ascribed to an interplay of several factors and experimental conditions, such as the amount of adsorbed sample, the presence of a few patches on the SLB, the surface roughness of individual sensors, and variations in working temperatures and, thus, in the lipid structure and fluidity.

**Aqueous buffer type impact on SALBs**. In the case of SiO_2_, which gives overall better SALB results than Au, the dependence of average frequency and dissipation shifts on the type of buffer and temperature is seen in Figure 6C,D, respectively. Here, no significant dependence of the final frequency and dissipation shifts on the buffer type is observed. However, the best quality of SLBs is obtained again at the liquid-disordered phase, at 52 °C. The frequency shifts range are (i) in the case of HEPES −24.9 ± 0.5 Hz (DPPC) and −26.3 ± 0.9 Hz (DMPC), (ii) in the case of Tris −23.3 ± 0.3 Hz (DPPC) and −24.3 ± 0.8 Hz (DMPC), and (iii) in the case of Tris-EDTA −20.6 ± 0.4 Hz (DPPC) and −22.3 ± 0.3 Hz (DMPC). The dissipation values are small, and range from (0.3 ± 0.1) × 10^−6^ to (0.6 ± 0.1) × 10^−6^ in all cases. The SALB formation in Tris-EDTA apparently yields for both zwitterionic lipids, DMPC, and DPPC, a slightly smaller adsorption of mass associated with the relative amplitude of frequency shifts. Very likely, EDTA intercalates between the bilayers, as it has been proposed in previous studies [48,49], and increases the average effective surface area per lipid molecule. At the same time, the very small dissipation suggests that it does not increase the presence of defects. Our results indicate that, when required for certain experiments, the addition of EDTA not only maintains the quality of the SALBs but also enhances the visualization of the transition peak. However, this conclusion is valid for modest concentrations such as 5 mM used in the present study. At much higher concentrations (~150 mM), a stronger impact has been reported on the area per molecule and the organization of zwitterionic lipid layers [49].

From the values presented in the previous paragraph, it appears that HEPES at 52 °C on SiO_2_ yields slightly better values for both lipids compared to Tris and Tris-EDTA. However, regarding the detection of melting the situation is reversed; the melting cannot be probed in the case of HEPES, whereas it is detectable in Tris (DMPC) and Tris-EDTA (DMPC and DPPC), as shown in Figure 5B,D. The detection of melting in single SLBs requires time-consuming measurements, using surface-sensitive techniques [57,58]. In the case of QCMD, the strong influence of the substrate coupled to the adjacent bilayer, already shown to affect even the melting of SLVs [56], has an impact on the detection in the case of SLBs. Our results suggest that in the case of Tris and even more in the case of Tris-EDTA, there is an apparent screening of the substrate influence, allowing the detection of lipid melting. The phase transition peak, albeit weak, occurs within a narrow temperature range. This indicates that, unlike what is observed for SLVs using QCM-D [56] and for SLBs on hydrophilic surfaces using AFM and neutron scattering [58,59], the bilayer melting is not decoupled between the two leaflets. AFM studies, performed on mica substrates, have reported variable influence of different aqueous buffers and ions on the stability and mechanical properties of SLBs [58,60]. Combined QCM-D and AFM investigations on identical substrates and lipids, changing only one experimental parameter (e.g., buffer type, ionic strength) at a time, could shed light on the subtle nature of these differences.

**Figure 7:**
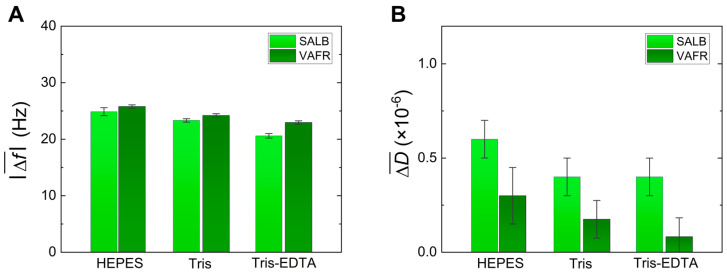
Comparison of the QCM-D final (absolute) values of the frequency (panel (**A**)) and dissipation (panel (**B**)) shifts obtained in the case of DPPC SALBs and DPPC SLBs produced by the VAFR method, performed on SiO_2_ surfaces at 52 °C.

**Comparison of SALB and VAFR methods**. In Figure 7, a comparison is shown between the best-obtained SALBs and SLBs produced by the VAFR process (50 nm extruded vesicles) of DPPC at 52 °C on SiO_2_ using the three buffers: HEPES, Tris, Tris-EDTA. As seen in Figure 7A, the frequency shifts, ranging from −20.6 Hz to −24.9 Hz for the SALB and from −22.9 Hz to −24.8 Hz for VAFR (errors within ±1 Hz), are very close to each other, as well as reasonably close to the value of complete homogeneous SLBs [54]. Therefore, both methods yield SLBs of similar quality, with only a mild decrease in both frequency and dissipation shifts when moving from HEPES to Tris and, finally, to Tris-EDTA.

## 5. Conclusions

By means of QCM-D, we have investigated the influence of adsorption temperature (15 °C, 52 °C), the type of aqueous buffer (HEPES, Tris, Tris-EDTA), and the surface of the solid substrate (Au, SiO_2_), on the quality of SALB formation. Moreover, we have provided a brief comparison between the best-obtained SALBs and SLBs produced via VAFR. For all our measurements, we have strictly adhered to the literature standards regarding sample preparation, flow rates, and concentrations [36,38,39].

Regarding the surface of the solid substrate, SALBs yield more consistent results (almost independent of the lipid phase during the adsorption) in the case of SiO_2_ with respect to Au. For the latter, an improvement of the bilayer homogeneity, reflected in both frequency and dissipation values, is obtained at higher temperatures. The quality of the so-produced SLBs is generally better when the adsorption is performed at temperatures corresponding to the liquid-disordered phase. When the SALB is formed in the gel phase, it appears more sensitive to the choice of surface, especially in the case of Au. The influence of the buffer on the quality of the SALB is mild; however, it has a clear impact on the detection of the lipid melting being pronounced for Tris-EDTA. Note that the sensing of lipid melting by a purely non-destructive method such as QCM-D could be beneficial in certain experiments, e.g., when sequential thermal cycling or repetition of measurements are necessary. The influence of EDTA on the lipid organization has also a mild effect on the SALB; namely, a smaller adsorbed mass without induction of defects. Therefore, the addition of modest amounts of EDTA, without degrading the quality of the SALB, facilitates the QCM-D detection of the melting transition in thin lipid bilayers. Finally, for adsorption on SiO_2_ at temperatures corresponding to the liquid-disordered phase, SALBs consistently produce bilayers of essentially identical quality as VAFR, without being subject to as many constraints and large timescales (i.e., limited substrates, extrusion of vesicles, time-consuming evaporation of chloroform and hydration) as the latter.

## Data Availability

The authors are willing to provide the raw data of this work at any time, upon reasonable request.

## Supplementary Materials

The following supporting information can be downloaded at: https://www.mdpi.com/article/10.3390/s24186093/s1, Figure S1: The influence of the solid surface on the DMPC SALB formation, performed at 15 °C, is shown here for Au (yellow curves) and SiO_2_ (grey curves). The frequency and the dissipation changes of the 3rd overtone are shown for both cases on the left panel A and the right panel B, respectively. The IPA injection step occurred after the HEPES baseline and the lipid injection was cut (albeit shown for DPPC in the main manuscript) in order to enhance the visibility of the final shifts. Figure S2: The influence of adsorption temperature on DMPC SALBs for Au (left panels A) and SiO_2_ (right panels B) substrates; the frequency and dissipation changes are shown for the 3rd overtone at the top and bottom layers of each panel, respectively. The dashed lines serve as guides to the eye, denoting the QCM-D frequency shifts corresponding to a complete and homogeneous SLB surface coverage. Table S1: Corresponding hydrated masses per unit area of lipid layers are displayed in Figure 2 and Figure S1 and formed by the solvent-exchange process at 15 °C. The masses were calculated via the Sauerbrey model (for SiO_2_), or the viscoelastic model (for Au), and correspond to the mass per unit area of the lipids with their coupled hydration layer. The fits were performed on curves obtained after the SALB process, in the HEPES buffer, at 15 °C.
