# Supplementary material for: Assessing the Quality of Solvent-Assisted Lipid Bilayers Formed at Different Phases and Aqueous Buffer Media: A QCM-D Study
Source: Sensors (Basel). 2024 Sep 20;24(18):6093. doi: 10.3390/s24186093 (PMC11435612; doi:10.3390/s24186093)
Supplement: Supplementary file 1 [file sensors-24-06093-s001.zip › sensors-3194222-supplementary.pdf]

sensors

MDPI

Assessing the quality of solvent-assisted lipid bilayers formed at different phases and aqueous buffer media: A QCM-D study

Marta Lavrič, Laure Bar*, Martin E. Villanueva, Patricia Losada-Pérez, Aleš Iglič, Nikola Novak, and George Cordoyiannis*

Supplementary Material

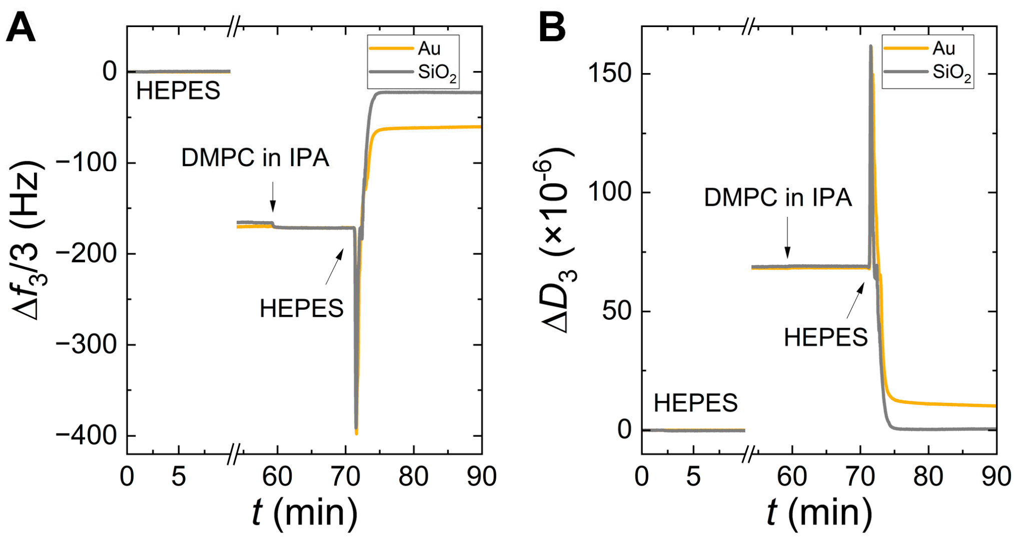

Figure S1. The influence of the solid surface on the DMPC SALB formation, performed at 15 °C, is shown here for Au (yellow curves) and SiO_2 (grey curves). The frequency and the dissipation changes of the 3rd overtone are shown for both cases on the left panel A and the right panel B respectively. The IPA injection step occurring after the HEPES baseline and the lipid injection was cut (albeit shown for DPPC in the main manuscript) in order to enhance the visibility of the final shifts.

The average reported final values, obtained for a DMPC bilayer formation at 15° C, are Δf = -51 ± 5 Hz and ΔD = (9 ± 1) × 10^{-6} on Au-coated sensors, which reflects the presence of defects within the layer, including a few non-ruptured vesicles. The corresponding values obtained on SiO_2-coated sensors are Δf = -21 ± 1 Hz and ΔD = (0.5 ± 0.1) × 10^{-6}, characteristic of a thin organized supported layer.

Table S1. Corresponding hydrated masses per unit area of lipid layers displayed on Figures 2 and S1, and formed by solvent-exchange process at 15°C. The masses were calculated via the Sauerbrey model (for SiO_2), or the viscoelastic model (for Au), and correspond to the mass per unit area of the lipids with their coupled hydration layer. The fits were performed on curves obtained after the SALB process, in HEPES buffer, at 15°C.

**surface**	**lipid**	
	DPPC hydrated mass (ng/cm²)	DMPC hydrated mass (ng/cm²)
**Au**	814 ± 50	646 ± 58
**SiO_2**	396 ± 33	382 ± 19

From the values of this table, one can see that in the case of Au there is significantly larger hydrated mass for both lipids, related to the presence of unruptured vesicles and extra patches, as already discussed in the manuscript.

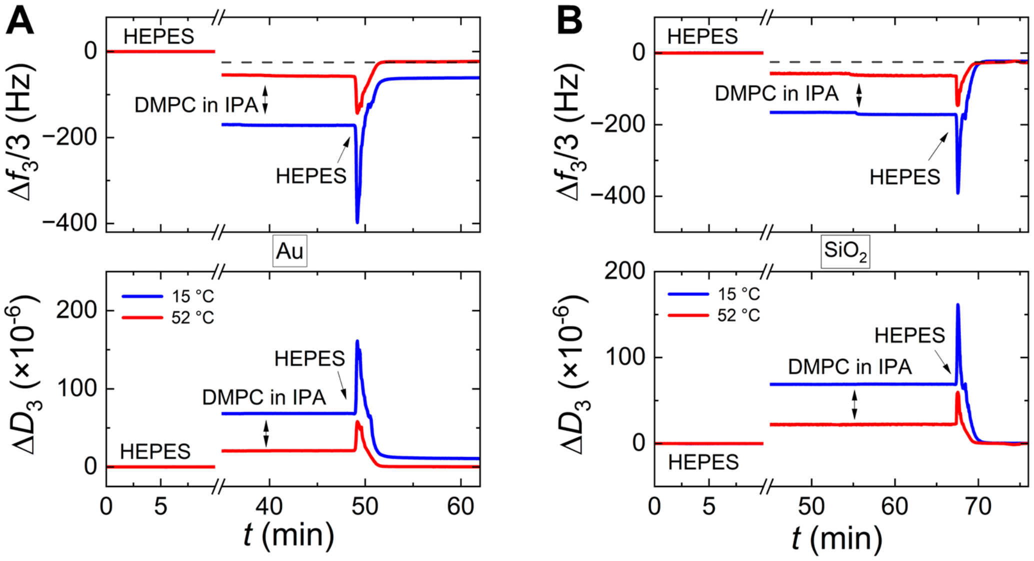

Figure S2. The influence of adsorption temperature on DMPC SALB for Au (left panels A) and SiO_2 (right panels B) substrates; the frequency and dissipation changes are shown for the 3rd overtone at the top and bottom layers of each panel respectively. The dashed lines serve as guides to the eye, denoting the QCM-D frequency shifts corresponding to a complete and homogeneous SLB surface coverage.

On SiO_2 surfaces, the SALB process produces complete defect-free bilayers, independently of the adsorption temperature. In the case of Au-coated surfaces, working at temperatures corresponding to the liquid-disordered lipid phase strongly improves the quality of the bilayer and prevents the formation of vesicle-containing SALB. On Au surfaces, the average reported final values obtained for a DMPC bilayer formation in liquid phase are $\Delta f = -24.4 \pm 0.2$ Hz and $\Delta D = (0.3 \pm 0.2) \times 10^{-6}$, whereas in the gel phase $\Delta f = -51 \pm 5$ Hz and $\Delta D = (9 \pm 1) \times 10^{-6}$.
